# Development and Validation of an Instrument for the Detection of Early Traumatic Experiences (ExpTra-S) in Patients With Psychosis

**DOI:** 10.3389/fpsyg.2020.528213

**Published:** 2020-10-29

**Authors:** Mercedes Paino, Nuria Ordóñez-Camblor, Eduardo Fonseca-Pedrero, Leticia García-Álvarez, Juan Pablo Pizarro-Ruiz

**Affiliations:** ^1^Department of Psychology, University of Oviedo, Oviedo, Spain; ^2^Health Sciences, University of Burgos, Burgos, Spain; ^3^Educational Sciences, University of La Rioja, Logroño, Spain; ^4^Center for Biomedical Research in the Mental Health Network (CIBERSAM), Madrid, Spain; ^5^Educational Sciences, University of Burgos, Burgos, Spain

**Keywords:** psychosis, early traumatic experiences, psychopathology, self-report, psychometric properties

## Abstract

The risk of the appearance of psychosis may reflect the existence of an underlying vulnerability, which may be influenced by environmental factors such as early traumatic experiences. This means that in clinical practice, the assessment of and approach to previous traumatic events is important in persons with psychotic disorders. The psychometric assessment of trauma has advanced considerably in recent years; however, there is no instrument that has been constructed and validated specifically for the evaluation of early traumatic experiences in the clinical population with psychosis. The main goal of this study was to present the construction and validation process of the *Screening of Early Traumatic Experiences in Patients with Severe Mental Illness* (ExpTra-S). The sample consisted of 114 patients who had experienced at least one psychotic episode (*M* = 35.5 years of age; *SD* = 9.26) and a comparison group of 153 young adults (*M* = 20.8 years of age; *SD* = 1.8). The factor analysis revealed an essentially one-dimensional structure. The ExpTra-S was associated with the positive dimension of the psychosis phenotype but not with the negative or affective dimensions, or subjective experiences. No items displayed differential functioning for sex and age. The ordinal alpha for the total score was 0.96. The patients with psychosis had a higher score for early traumatic experiences in comparison with the non-clinical group. The results obtained showed that the measuring instrument developed, the ExpTra-S, is a brief, simple, and useful measuring instrument for assessing the presence of early traumatic experiences in patients with severe mental illness.

## Introduction

Different research shows that people who have suffered abuse have worse overall mental health, with a greater presence of psychiatric symptoms and disorders (Sweeney et al., 2015; Park et al., 2016; Sonu et al., 2019). Patients with mental health problems have a 34–53% history of physical and sexual abuse in childhood (Alarcón et al., 2010). Childhood trauma has been associated with the appearance of a variety of psychopathological disorders such as mood and anxiety disorders, posttraumatic stress disorder, personality disorders, dissociative disorders and substance use disorders, among others (Kim and Lee, 2016; Aas et al., 2017; Gidzgier et al., 2019).

Currently, there is a great deal of research showing childhood trauma—physical, sexual, and emotional abuse and neglect—as a risk factor for psychotic disorder (De Loore et al., 2007; Trauelsen et al., 2015). In this regard, various studies have found that traumatic experiences can play a causal role in the development of psychotic disorders (Read et al., 2005; Thompson et al., 2014). A meta-analysis by Varese et al. (2012) estimated a 3-fold increase in risk for psychosis after exposure to childhood traumatic events.

Similarly, a relationship has been observed between different psychotic symptoms and traumatic experiences. The presence of the positive symptoms of schizophrenia has been associated with a previous history of trauma (Ordóñez-Camblor et al., 2014; Duhig et al., 2015), with paranoid delusions and hallucinations being the symptoms that have most been associated with early traumatic experiences (Rhodes et al., 2018; Luhrmann et al., 2019). Trotta et al. (2015) conduct a meta-analysis and conclude that childhood traumatic experiences increase by 1.8 the probability of having hallucinations and delusions in people without a diagnosis of psychosis (OR 1.76, 95% CI 1.19–2.32) and by 1.5 in diagnosed persons (OR 1.55, 95% CI 0.32–2.77). According to Garety et al. (2001), vulnerability to psychosis (from a biopsychological approach) would lead people who experience trauma to develop maladaptive cognitive and emotional functioning that would also lead to delusions and hallucinations (positive symptoms). The traumatic experiences lived, together with the cognitive alterations of vulnerable people, would lead to ambiguous and unstructured sensory inputs that are not recognized and experienced as strange. These abnormal experiences are associated with emotional changes that are important to the person and cause the search for an explanation of their cause. Thus, an early traumatic experience increases that cognitive vulnerability generating lasting negative schemes about themselves and the world, which together with the emotional changes, would feed delusions and hallucinations. Kuipers et al. (2006) argue that trauma and adversity affect both information processing and emotional processing, leading to experiences that are then misinterpreted and assessed as symptoms of psychosis.

The importance of the relationship between trauma and psychopathology has meant that in recent years the development of questionnaires for the measurement of trauma has increased. However, there is no instrument that has been constructed, validated and normalized specifically for the evaluation of traumatic experiences in the clinical population with psychosis. This is important, because it has been found that patients with psychotic disorders often have lower IQ and neuropsychological performance than the general population (Reichenberg and Harvey, 2007). The use of psychopharmacological treatments and the course of the disorder itself have been associated with increased cognitive impairment in psychosis (Veselinovic et al., 2013). It is therefore necessary for the assessment instruments to be simple, to use clear and understandable language and not to be too long. Internationally, most of the existing tools for assessing early traumatic experiences have little or no psychometric support (Donald, 2012). Also, the vast majority of the measurement instruments that have been developed to evaluate this do not incorporate the new statistical procedures such as item response theory (IRT) or differential item functioning (DIF) (Ordóñez-Camblor et al., 2016). Similarly, there are few instruments that are available in the specialist literature that evaluate the distress associated with these experiences (Ordóñez-Camblor et al., 2016). This is important since, from a clinical point of view, the way in which the patient processes and manages the trauma is critical in working through the traumatic experiences. In this context, the main purpose of this study was to develop a short, simple, and useful measuring instrument for assessing the frequency and the distress of the early traumatic experiences, often found in patients with psychosis.

## Materials and Methods

### Participants

In this research, two samples of participants were used: patients with psychotic disorders and a comparison group of young, non-clinical adults. The clinical sample consisted of 114 patients, 82 men (71.9%) who had experienced, at some point in their lives, at least one psychotic episode. The selection of participants was conducted in different mental health centers in the Principality of Asturias, Cantabria, and Catalonia, three regions of northern Spain. Among the entire population served in the centers, patients who were at that time in treatment for a schizophrenia spectrum disorder according to the Diagnostic and Statistical Manual of Mental Disorders (DSM-5, American Psychiatric Association [APA], 2013) were selected; 71 (62.3%) patients had a diagnosis of schizophrenia, 19 (16.7%) of brief psychotic disorder, 7 (6.1%) of schizoaffective disorder, 7 (6.1%) of bipolar disorder with psychotic symptoms, 5 (4.4%) of delusional disorder, 3 (2.6%) of schizophreniform disorder, and 2 (1.8%) of schizotypal disorder. The mean age was 35.5 years (*SD* = 9.26), the age range varying between 14 and 52 years. The average number of psychotic episodes was 2.68 (*SD* = 1.81) with an average of 1.48 hospitalizations (*SD* = 1.72). The average age of the first episode was 25 (*SD* = 7.21), ranging from 9 to 45. Table 1 shows the sociodemographic and clinical characteristics of the sample of patients with psychosis.

**TABLE 1:** Sociodemographic and clinical characteristics of the sample of patients with psychosis.

**Variables**	**Men (*n* = 82)**	**Women (*n* = 32)**	**Total (*n* = 114)**
Age, Mean (SD)	35.04 (9.05)	36.78 (9.81)	35.53 (9.26)
**Education**			
Elementary school, *n* (%)	20 (24.4)	8 (25.0)	28 (24.6)
Middle school/FP1*, *n* (%)	23 (28.6)	5 (15.6)	28 (24.6)
High school/FP2*, *n* (%)	24 (29.3)	10 (31.3)	34 (29.8)
University, n (%)	15 (18.3)	9 (28.1)	24 (21.1)
**Employment situation**			
Employed, *n* (%)	21 (25.6)	8 (25.0)	29 (25.4)
Unemployed, *n* (%)	61 (74.4)	24 (75.0)	85 (74.6)
**Nationality**			
Spanish, *n* (%)	79 (96.3)	29 (90.7)	108 (94.7)
Other**, *n* (%)	3 (3.7)	3 (9.3)	6 (5.4)
**Treatment**			
Pharmacological, *n* (%)	54 (65.9)	18 (56.3)	72 (63.2)
Combined, *n* (%)	28 (34.1)	14 (43.8)	42 (36.8)
**Personal psychiatric antecedents**			
Yes, *n* (%)	35 (42.7)	19 (59.4)	54 (47.4)
No, *n* (%)	47 (57.3)	13 (40.6)	60 (52.6)
**Family psychiatric antecedents**			
Yes, *n* (%)	25 (30.5)	9 (28.1)	34 (29.8)
No, *n* (%)	57 (69.5)	23 (71.9)	80 (70.2)

*FP1 and FP2 are two levels of vocational training—basic and advanced. **Other nationality (Romanian, Cuban, Argentine, Moroccan, Russian, and Mexican).

As a comparison group, a sample of non-clinical young adults was selected, also incidentally, ultimately making up a total of 153 university students, 122 women (79.7%), belonging to different social and health sciences courses at the University of Oviedo and the University of La Rioja. The mean age of participants was 20.8 years (*SD* = 1.8), the age range varying between 18 and 30 years.

### Instruments

*Screening of Early Traumatic Experiences in Patients with Severe Mental Illness* (ExpTra-S): Developed by our team, this is a short measuring instrument for assessing early traumatic experiences in clinical samples of patients with psychosis. It consists of two scales, one of frequency and one of distress. The frequency scale is composed of a total of 18 items in *Likert* response format using four categories (0 “never,” 1 “sometimes,” 2 “frequently,” and 3 “almost always”). The presence of early traumatic experiences is evaluated through 17 questions about different types of child abuse, namely sexual abuse, physical and psychological abuse, and physical and emotional neglect, one last item being added that refers to any other type of traumatic event that may have occurred, about which there have been no previous questions, and which has generated distress in the patient. It also incorporates the scale of distress associated with these experiences, which comprises 18 items in *Likert* response format using four categories (1 “no distress” to 4 “great distress”). This scale of distress levels should only be answered if the traumatic experience is present at least “sometimes” on the frequency scale.

*Community Assessment of Psychic Experiences-42* (CAPE) (Stefanis et al., 2002): This is a self-report which assesses psychotic experiences with regards to their positive, negative and emotional aspects. The CAPE consists of 42 items that assess the following dimensions of psychotic symptoms: positive, negative and depressive. Each question is answered in a Likert-type format four points, ranging from “never” (1) to “almost always” (4). If the participants choose the response options “sometimes”, “often” or” almost always”, they must indicate the degree of distress that the experience causes them, also on a Likert-type scale with four categories (1, “no distress” to 4 “great distress”). In this study, the validated and adapted into Spanish version by Fosenca-Pedrera et al. was used. Internal consistency values for the three dimensions of the CAPE-42 ranged between 0.84 and 0.93 (Fonseca-Pedrero et al., 2012).

*The Frankfurt-Pamplona Scale of Subjective Experiences, EES, Spanish version* (Süllwold, 1986; Cuesta et al., 1995): The EES consists of 18 items, through which the subjective complaints of cognitive deficits are evaluated in patients with psychosis. The answers are expressed using a *Likert* scale with five possible answers (”never” 0 to “almost always” 4). The higher the score, the greater the presence and frequency of subjective complaints of cognitive deficits. The scale has adequate psychometric properties, showing high internal consistency (Cronbach’s alpha 0.91) and a one-dimensional internal structure (Cuesta et al., 1995).

### Procedure

#### Construction of the ExpTra-S

The construction process of the test was conducted according to the international guidelines for the construction of measuring instruments (American Educational Research Association, 1999; Wilson, 2005; Downing, 2006; Schmeiser and Welch, 2006) and following a series of steps that would ensure the construction process was undertaken systematically and rigorously (Muñiz and Fonseca-Pedrero, 2019).

In generating the items of the ExpTra-S, the proposed guidelines for the construction of multiple-choice items were taken into consideration (Muñiz et al., 2005; Moreno et al., 2006). The content validity of the items was assured, based on the judgment of experts and an exhaustive review of the various self-reports that exist in the literature for the evaluation of traumatic experiences in adults. The main self-reports that were used as models for the construction of the ExpTra-S item bank were: (a) *The Sexual Abuse Exposure Questionnaire* (SAEQ) (Rodríguez et al., 1997); (b) *The Childhood Trauma Questionnaire* (CTQ) (Bernstein et al., 1994) or its abbreviated version (CTQ-SF) (Bernstein et al., 2003); (c) *The Trauma History Questionnaire* (THQ) (Green, 1996); and (d) questions about traumatic experiences used in research in the study of the psychotic phenotype (Wigman et al., 2011; Kelleher et al., 2013).

The initial set of items was developed based on: (a) the adaptation and translation of items from other self-reports for the assessment of traumatic experiences in adults, following the international guidelines for translating and adapting tests (Muñiz et al., 2013; Hernández et al., 2020); (b) the adaptation and/or modification of items: items from other self-reports in which changes had been made so that they could be understood by patients; and (c) the construction of new items: new elements were produced following the guidelines mentioned above for the construction of multiple-choice items. After the judgment of experts and with the aim of obtaining a screening instrument on traumatic experiences that was brief and simple in language, a version of the ExpTra-S was configured with 5 items from the CTQ (items 1, 2, 3, 4, and 11), 9 new items, and 4 modified items following the guidelines on the language to be used (3 items were from the CTQ and 1 from the THQ).

For the construction and validation of the questionnaire, two pilot studies were conducted, one qualitative and one quantitative, as well as a final field study. The qualitative pilot study was conducted in order to examine the overall functioning of the questionnaire, the detection of any type of error and the pooling of possible suggestions to improve the items or the questionnaire. The quantitative pilot study was conducted on a non-clinical sample in order to pare down the initial item bank. For this purpose, a group of approximately 15 people was formed to which this initial version of the ExpTra-S was applied, where each person made their comments aloud. A two-hour session was held in order to follow a standardized process, and a series of phases were established to guide the structuring of the session: Phase 1: Introduction to the session, general theme of the study and how to proceed during the session, Phase 2: Reading of the items individually in order to detect the presence of strange or difficult to understand words., Phase 3: Reading of the items aloud by the coordinator and sharing of the individual impressions, Phase 4: Individual evaluation of the degree of comprehensibility of the different items and suggestions for their improvement (they were asked two questions regarding *what they understood was being asked about that particular item*, and *whether they thought that item was easy or difficult to understand*. If they found the item difficult to understand, they should indicate how they would reformulate it to make it more comprehensible). Phase 5: Integration of the information, approval by the participants of the main changes to be introduced. The empirical criteria established for the elimination of items were: (1) items with discrimination indices below 0.20; (2) items whose factor load was not saturated in the general factor or did not fit an essentially one-dimensional solution; and (3) items with factor loadings less than 0.25. No item was eliminated from the measuring instrument according to the statistical criteria.

#### The Administration of the ExpTra-S

The administration of the questionnaire to the sample of persons with psychosis was performed individually during a clinical session. Contact with patients was conducted through the mediation of their psychiatrists/psychologists from the mental healthcare centers. All participants gave their informed consent voluntarily to participate. The administration to the group of non-clinical young adults was conducted collectively, in groups of 10 to 30 participants, during class hours, and always under the supervision of a researcher. This research was approved by the Ethics Committee of Clinical Research of the University Central Hospital of Asturias (HUCA).

### Data Analysis

To carry out the study of the psychometric properties of the ExpTra-S, we proceeded to the study of obtaining validity evidences and reliability. First, the descriptive statistics of the items were calculated and an analysis of the internal structure of the ExpTra-S scores was conducted, using an exploratory factor analysis at the item level, on the polychoric correlation matrix. The estimation method was unweighted least squares with subsequent Promin rotation. The procedure for determining the number of dimensions was the optimal implementation of parallel analysis (Timmerman and Lorenzo-Seva, 2011).

Second, evidence based on relations to other variables was obtained by analyzing the Pearson correlations between the total score of the ExpTra-S, the dimensions of the CAPE-42 and the total score of the EES. Third, the differential item functioning (DIF) was analyzed according to sex and age. The generalized Mantel-Haenszel test (GMH) (Mantel and Haenszel, 1959) was used in this study, specifically *the Generalized Ordinal MH statistic* - QGMH (2). The level of statistical significance was set at *p* < 0.01. The Mantel-Haenszel procedure is among the most widely used for assessing DIF, due to its simplicity of calculation and interpretation. Fourth, the reliability of the total score was examined using ordinal alpha (Elosúa and Zumbo, 2008) and item response theory, through the information function. Finally, an analysis of covariance (ANCOVA) was conducted between the patient group and the non-clinical group (covariates sex and age), as well as a mean comparison by sex of the total ExpTra-S score.

For the data analysis, the programs used were SPSS 18 (Meulman and Heiser, 2009), FACTOR 9.0 (Ferrando and Lorenzo-Seva, 2017), GHMDIF (Fidalgo, 2011) and Mplus 5.2 (Muthén and Muthén, 2007, 2010).

## Results

### Descriptive Statistics and Prevalence of Traumatic Experiences

Table 2 shows the descriptive statistics related to the mean, standard deviation, asymmetry, kurtosis, factor loadings and estimated communalities for the distribution of items of the ExpTra-S, in the sample of patients with psychosis. The percentage of patients with psychosis who responded affirmatively [categories “sometimes” (1), “often” (2), or “almost always” (3)], to any of the items on the frequency scale ranged between 10.5% (item 4) and 52.6% (item 18). Of the patients with psychosis, 80% reported having experienced early traumatic experiences, compared with 48.4% of the group of non-clinical young adults. If the range is restricted and only those patients who responded “often” or “almost always” in the response options of the frequency scale of the ExpTra-S are selected, the percentage decreases (Table 3).

**TABLE 2:** Descriptive statistics and exploratory factor analysis for the items of the ExpTra-S in the clinical sample.

**Items**	**Mean**	***SD***	**Asymmetry**	**Kurtosis**	**Factorial load**	**Communality**
1	0.18	0.45	2.59	6.30	0.67	0.45
2	0.28	0.59	2.25	5.06	0.80	0.64
3	0.29	0.49	1.38	0.89	0.75	0.56
4	0.12	0.38	3.26	10.76	0.77	0.59
5	0.25	0.59	2.81	8.52	0.76	0.58
6	0.14	0.40	2.92	8.45	0.56	0.32
7	0.14	0.37	2.60	6.35	0.64	0.41
8	0.45	0.86	1.93	2.68	0.82	0.68
9	0.27	0.54	1.87	2.64	0.73	0.53
10	0.32	0.67	2.18	4.35	0.88	0.77
11	0.46	0.83	1.85	2.59	0.70	0.48
12	0.46	0.73	1.54	1.71	0.82	0.67
13	0.53	0.85	1.61	1.73	0.80	0.65
14	0.58	0.88	1.57	1.68	0.69	0.47
15	0.21	0.63	3.25	10.22	0.78	0.61
16	0.35	0.79	2.17	3.58	0.63	0.40
17	0.39	0.81	2.16	3.89	0.84	0.71
18	0.70	0.79	0.92	0.26	0.48	0.23

**TABLE 3:** Percentages of patients with psychotic disorders responding to ExpTra-S response options.

**Item**	**Never**	**Sometimes**	**Frequently**	**Almost always**
Were you beaten so badly by your parents or a close relative that you had to go to the doctor or hospital?	85.1	12.3	2.6	0
A family member hit you so hard that it left bruises or marks on your body?	78.1	16.7	4.4	0.9
Were you punished by being hit with a hard object like a belt, a stick or a rope?	72.8	25.4	1.8	0
Were you hit so hard that someone else, like a teacher, a neighbor or a doctor, noticed?	89.5	8.8	1.7	0
Did someone physically abuse you?	81.6	14.0	2.6	1.8
Did someone sexually abuse you?	87.7	10.5	1.8	0
Did someone try to force you (against your will) to do things related to sex?	86.8	12.3	0.9	0
When you were a child, did any family member regularly and repeatedly insult you?	73.7	14.0	6.1	6.1
Did your parents or close relatives tell you it would be better if you had not been born?	77.2	18.4	4.4	0
Were the comments your parents made about your behavior insulting or hurtful, even making you cry?	77.2	14.9	6.1	1.8
How often have you had the feeling that your parents or close relatives hated you?	70.2	18.4	6.1	5.3
Were you emotionally abused?	66.7	22.8	8.8	1.8
Have you felt hated or rejected by your family?	65.8	21.1	7.9	5.3
Have you felt insecure, unprotected and unloved by your family?	61.4	26.3	5.3	7.0
When you were sick, didn’t they take you to the doctor out of neglect or carelessness?	87.7	6.1	3.5	2.6
How often did one of your parents drink and get drunk and not take care of you or your siblings?	80.7	7.0	8.8	3.5
Have your parents or close relatives told you that you might be a failure in life?	75.4	14.9	4.4	5.3
Have you ever experienced a traumatic event (e.g., accident and loss of a close family member) that you have not been asked about before, that you feel was important, and that caused you discomfort?	47.4	37.7	12.3	2.6

### Validity Evidence Based on the Internal Structure of the ExpTra-S

In order to examine the internal structure of the ExpTra-S scores, an exploratory factor analysis was performed at the item level, on the polychoric correlation matrix. The Bartlett’s sphericity index was 1204.6 (*p* < 0.001) and the Kaiser–Meyer–Olkin value was 0.85. The analysis of the underlying dimensionality of the ExpTra-S scores revealed an essentially one-dimensional structure. The optimal implementation of the parallel analysis at 95% recommended the extraction of a general factor that explained 57.06% of the total variance (*eigenvalue* of 10.27). The RMSR value (Root Mean Square Residual) was 0.09, the GFI (Goodness-of-Fit Index) was 0.95, and the Kelly Criterion was 0.09.

Table 2 shows the factor loadings and estimated communalities for the essentially one-dimensional solution. As can be seen, all factor loadings were above 0.30; the percentage of variance explained by the first factor was high and the resulting goodness-of-fit indices were adequate. From these data, it is considered appropriate to construct a total score that reflects the latent dimension or variable of early traumatic experiences.

### Evidence Based on Relations to Other Variables

Evidence regarding the external variables was obtained by analyzing the Pearson correlations between the total score of the ExpTra-S, the psychotic-experience dimensions of the CAPE-42 and the total EES (subjective experience) score. The results indicated that the total ExpTra-S scores only correlated moderately with the score of positive symptoms of the CAPE-42 (Frequency *r*_*xy*_ = 0.393 and Distress *r*_*xy*_ = 0.326; *p* < 0.01). Thus, traumatic experiences—both in their frequency and their distress—are associated with the positive symptoms of the psychotic phenotype, but not with the negative or depressive symptoms, nor with the subjective experiences, measured with the EES.

### Differential Item Functioning According to Age and Sex

To analyze DIF by age (young and mature adults), we divided our sample into two groups, those with ages equal to or less than and those with ages greater than 35 years. The data indicated that none of the 18 items that make up the ExpTra-S showed differential functioning according to the age and sex of the patients, ensuring equity in the measuring process.

### Estimating the Reliability of the ExpTra-S Scores

**FIGURE 1:**
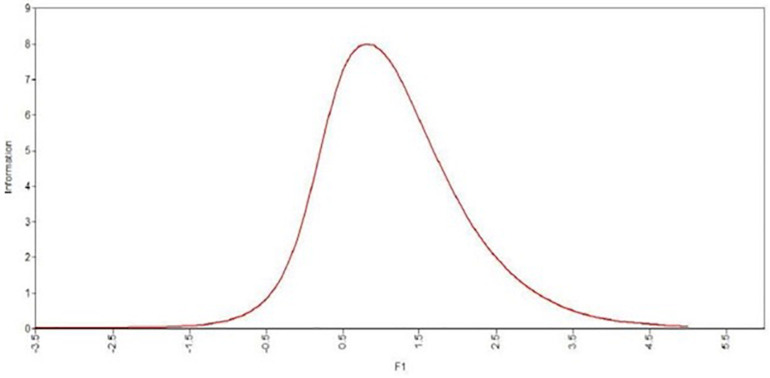
Information function of the *ExpTra-S.*

This analysis was conducted with the ordinal coefficient alpha. The alpha value was 0.96. All discrimination indices were higher than 0.30. The reliability of the scores was also estimated based on IRT. The resulting test information functioning in the clinical sample is presented in Figure 1. The highest degree of accuracy in estimating the latent trait was in the values ranging from 0.5 to 1.5. This result is of interest, because it is important in the clinical population of patients with psychosis to measure accurately the intermediate values of the latent construct and not the outliers.

### Differences in Mean Scores by Sex and Group

The mean scores of the ExpTra-S were compared, firstly, by sex in the clinical group and, secondly, between the group of patients with psychosis and the comparison group. In the sample of patients, no statistically significant differences were found according to sex in the mean total score of the ExpTra-S: *M*(*SD*)_*ma*__*le*_ = 5.75(7.0); *M*(*SD*)_*female*_ = 7.06(8.83); *t* = -0.829; *p* = 0.409. When the total mean score was compared by group controlling for sex and age, statistically significant differences were found (*M*(*SD*)_*c*__*omparison*_ = 1.48(2.60); *M*(*SD*)_*clinical*_ = 6.12(7.45); *F* = 6.300; *p* = 0.013, η*^2^* = 0.023). The group with psychosis had a higher score of traumatic experiences compared with the group of young adults. The effect size calculated by partial eta squared was high, indicative of the practical significance of the results.

## Discussion

The main objective of this study was to develop a measuring instrument adapted to patients with psychosis that would be a brief, simple and useful measure for evaluating the frequency and distress of early traumatic experiences. It is extremely important that this objective is achieved, since there is currently no specific measuring instrument for assessing this type of experience in patients with psychosis. Likewise, there are few measuring instruments that evaluate the distress the subjects experience from these traumatic experiences. It is worth mentioning that the ExpTra-S has been constructed based on substantive empirical criteria, which enables us to obtain greater evidence of validity based on the participants’ scores using the measuring instrument.

The ExpTra-S is a short measurement instrument consisting of 18 items with a simple and understandable writing. The use of a simple wording is important, especially if we consider that many patients with psychosis usually present some degree of cognitive deterioration (Chico del Río et al., 2013). Similarly, the importance of having measurement instruments that save time and effort should be mentioned, since the excessive length reduces the motivation of patients. In the literature, self-reports measuring early traumatic experiences in adults with more than 25 items can be found, such as the Childhood Trauma Questionnaire (CTQ) (Bernstein et al., 1994) or the Early Trauma Inventory (ETI) (Bremner et al., 2000).

The results of this study indicated that the ExpTra-S had adequate psychometric properties in this sample of patients with psychosis. The ExpTra-S scores have proven to be reliable in terms of internal consistency. Much evidence of validity has also been collected regarding early traumatic experience sin Spanish patients with psychosis. The estimation of the reliability showed a degree of internal consistency of 0.96, with all discrimination indices greater than 0.30.

The analysis of the internal structure of the ExpTra-S, performed by exploratory factor analysis, supported the one-dimensional nature of the construct. To date, the dimensionality of this construct has not been studied in depth. While several instruments have been developed that have methodologically incorporated a more sophisticated approach in the evaluation of childhood traumatic experiences in recent years, relatively little attention has been paid to the evidence of validity (Bernstein et al., 2003) with few studies providing information about the construct validity of the instruments used (Burgermeister, 2007).

The external validity was examined by analyzing the relationship of the two total scores on the ExpTra-S, with the scores on the CAPE-42 and the EES. Traumatic experiences, both in frequency and distress, are associated with the positive symptoms of the psychotic phenotype, but not with the negative or depressive symptoms, nor with the subjective experiences measured using the EES. These results converge with the data found in the previous literature. For example, Sheffield et al. (2013) studied the relationship between early traumatic experiences and auditory hallucinations, in 114 patients with psychosis. The results showed that patients with psychosis who had experienced auditory hallucinations reported a greater presence of sexual, physical and emotional abuse, compared to patients who had never had auditory hallucinations. With respect to the negative symptoms of psychotic disorders, previous studies indicate that the relationship between early traumatic experiences and negative symptoms is less clear (Gallagher and Jones, 2013; Akbey et al., 2019).

The analysis of differential item functioning (DIF) enabled us to ensure the measurement process was fair. In the process of constructing and validating the ExpTra-S, a study of the DIF was conducted according to the sex of the participants. There have been few studies investigating DIF in this study area, and yet it is a very important aspect of the validity of the data (American Educational Research Association, 1999). The presence of DIF assumes that the probability of getting the correct answer depends not only on the level of the person in the variable being measured, but it is also conditioned by their membership of a particular social, cultural, linguistic, etc. group, which generates a lack of metric equivalence between the scores (Elosúa, 2003).

With regard to the presence of traumatic experiences by sex and group of origin, the data indicate a greater presence of early traumatic experiences in patients with psychosis, with no differences according to sex. These data are consistent with findings that suggest that patients with psychotic disorders often have a more severe history of childhood abuse than individuals without psychiatric disorders (Matheson et al., 2013; Mauritz et al., 2013; Church et al., 2017). With regard to sex, previous studies have found no statistically significant differences in the prevalence of early traumatic experiences between male and female patients with psychotic disorders (Velthorst et al., 2013; García et al., 2016).

When interpreting the findings of this study, the following limitations should be borne in mind. First, the characteristics of the sample must be considered, consisting of people with psychosis who have a relatively good level of functioning, stabilized individuals undergoing treatment as outpatients; this means the sample is not representative of all people with psychotic disorders and the results cannot be generalized to patients whose disorders would presumably be more severe. Second, the retrospective nature of the traumatic experiences that have been collected from patients must be noted; although the effect of distress that these experiences has led to seems to confirm its occurrence, the omission of other experiences that are not accessible to the memory at the present moment cannot be ruled out. Third, there is a difference in the male-female ratio of the clinical group and the comparison group; this limitation may have minor relevance, however, as it has been confirmed that there are no significant differences between the sexes in the presence of early traumatic experiences. Fourth, we must not lose sight of the cross-sectional nature of this research, which means that it is not possible to establish causal inferences. And finally, one must take into account how different in characteristics the sample is from the patients group and the comparison group when interpreting the results. The comparison group was made up of college students with no serious medical history, mostly women and younger than the clinical sample. Although our results have controlled for gender and age in the comparison between the clinical and non-clinical groups, and men and women do not differ in the levels of reported traumatic experiences (Goldberg and Freyd, 2006), it should be noted that women report experiencing more emotional, psychological and sexual abuse than men, who report higher levels of trauma related to physical violence, not only during childhood but also during adulthood (Goldberg and Freyd, 2006; Fisher et al., 2009).

Therefore, for future work in this line, it would be interesting to study the differences in the results collected by this questionnaire adapted to people with psychosis with respect to others in common use (i.e., CTQ), since the superiority of ExpTra-S in this group of patients remains to be demonstrated. Moreover, it would be important to continue to obtain new validity evidences of the ExpTra-S as well as examining other psychometric properties such as the test-retest reliability. Similarly, it would be interesting to apply the ExpTra-S in patients with other mental disorders. Moreover, future studies should further examine the role of traumatic experiences in the development of psychotic disorders, both in clinical samples and in people that are at high risk of developing psychosis.

## Data Availability Statement

The datasets generated for this study are available on request to the corresponding author.

## Ethics Statement

The studies involving human participants were reviewed and approved by Ethics Committee of Clinical Research of the University Central Hospital of Asturias (HUCA). Written informed consent to participate in this study was provided by the participants’ legal guardian/next of kin.

## Author Contributions

MP, NO-C, EF-P, LG-Á, and JP-R contributed to the conception and design of the study. NO-C, EF-P, and JP-R organized the database. NO-C, EF-P, and JP-R performed the statistical analysis. MP and NO-C wrote the first draft of the manuscript. MP, NO-C, EF-P, and JP-R wrote the sections of the manuscript. All authors contributed to the manuscript revision, and read and approved the submitted version.

## Conflict of Interest

The authors declare that the research was conducted in the absence of any commercial or financial relationships that could be construed as a potential conflict of interest.
